# Magnesium- and biopolymer-stabilized ACC and ACP form the body-wall spicules of *Baptodoris cinnabarina* (Doridida, Gastropoda)

**DOI:** 10.1038/s41598-026-47236-9

**Published:** 2026-04-20

**Authors:** E. Griesshaber, C. Salas, J. D. Castro-Claros, A. Sancho Vaquer, E. Macías-Sánchez, X. Yin, G. P. Laurent, N. Nassif, T. Azaïs, A. Checa, W. W. Schmahl

**Affiliations:** 1 Department of Geo- and Environmental Sciences, Ludwig-Maximilians- Universität München, Munich, Germany; 2 Departamento de Biología Animal, Facultad de Ciencias, Universidad de Málaga, Málaga, Spain; 3 Departamento de Estratigrafía y Paleontología, Facultad de Ciencias, Universidad de Granada, Granada, Spain; 4 Institute of Parasitology and Biomedicine López Neyra, Spanish National Research Council, Granada, Spain; 5 Scientific Technology, Minhang District, Bruker, Beijing, Shanghai, 200233 China; 6 Laboratoire de Chimie de la Matière Condensée de Paris (LCMCP), Sorbonne Université, CNRS, Paris, France

**Keywords:** ACC, ACP, Phosphorous and magnesium distribution, Architectured biomaterials, Internalized skeleton, Lightweight scaffold, Biophysics, Chemistry, Materials science

## Abstract

Doridoidean nudibranch mollusks integrate into their integumentary system, thus, into their body-wall tissue, large quantities of Ca-carbonate spicules. We report the chemical and structural characterization of the body-wall spicules of the sea slug *Baptodoris cinnabarina* (Discodorididae, Doridida). The aim of the study was an in-depth characterization of the spicule mineral and of the soft tissue within the spicules. We achieved the latter by using a combination of imaging (TEM, SEM, micro-CT), diffraction (XRD, EBSD, SAED) and spectroscopical (ssNMR, EDS) techniques. We demonstrate that the spicule mineral consists of amorphous calcium carbonate (ACC), complemented with amorphous calcium phosphate (ACP). The amorphous minerals are very stable and do not crystallize, even not at prolonged electron beam irradiation. We distinguish structural and chemical differences between the rim and the core portions of the spicules. The spicule rim consists of an array of concentric organic laminae and is reinforced with ACC and ACP granules. At the spicule rim the organic laminae are closely positioned, while at the spicule core the laminae are widely spaced. The spicule rim is strongly enriched in phosphorous and has increased magnesium contents, while the spicule core is highly enriched in magnesium and has low phosphorous contents. We observe in the spicules a rather graded distribution of ACP, ACC, organic substance, phosphate and magnesium. We propose that magnesium, organic substance and the phosphorous of the ACP stabilize spicule ACC, both within the living organism as well as when the spicules are extracted from the soft tissue and are kept separate.

**Supplementary Information:**

The online version contains supplementary material available at 10.1038/s41598-026-47236-9.

## Introduction

Calcareous or siliceous entities, such as isolated spicules, needles, or granules, are incorporated into the connective tissue of many species and of many mineralizing invertebrate phyla. Some examples are: the sclerites of cnidarian corals, the ossicles of echinoderms, the spicules of sponges, the spicules and granules of molluscs, the spicules of rhynchonellate brachiopod lophophores and many more^1–13^.

For biomineralization studies heterobranch gastropod molluscs (Doridoidea) are of particular interest. Many taxa of the superfamily Doridoidea lost the capability to secrete mineralized tissue, thus, to form, e.g., an external mineralized shell or cover that protects the soft tissue^14^. However, species of the family Dorididae are specific, as the organisms that comprise the Dorididae do secrete mineralized elements. These are spicule-shaped, isolated, Ca-carbonate entities. These entities do not form external mineralized structures, e.g., a shell or a mineralized cover. The spicules are buried within the doridid integument of the dorsal body-surface. Hence, the spicules are within the soft tissue of the animal, are within the mantle, the foot and the rhinophores (the cephalic sensorial tentacles). In these parts of the body, large quantities of spicules accumulate and generate structured assemblies. The spicules remain within the soft tissue for the entire life of the organism^1,6,11,13,15–20^. Accordingly, for doridoidean heterobranchs, the mineralized hard tissue that protects the soft tissue of the animal is internal. It is not external, as is the case for other molluscs, e.g., for the aculifera (vermiform molluscs covered by an assembly of individual sclerites or sclerites and plates) and/or for the conchifera (higher-class molluscs covered by a continuous shell). As the doridoidean heterobranchs are not protecting their soft tissue with a mineralized cover, their mantle acts as the surface cover of the organism^17–20^. The doridid mantle is specific, as, in contrast to the mantle of the aculifera and of the conchifera, it is not only involved in secretory tasks, but also in many other functions^19–21^.

Alder and Hancock (1845–1855)^22^ observed, for the first time, the presence of calcified spicules within the integument of doridid gastropods. Odum (1951)^23^ reported their large abundance and suggested for the doridoidean species *Archidoris* that its spicules consist of amorphous calcium carbonate (ACC). Foale and Willan (1987)^16^ confirmed the large quantity of spicules in the doridoidean gastropod mantle, foot and gills and reported that the doridoidean dorsal surface is covered with specialized sensory tubercles, the caryophyllidia, and demonstrated that these as well are associated with assemblages of calcareous spicules.

The presence and distribution of the spicules within the doridoidean soft tissue is well known by now (e.g., Penney et al. (2018, 2020), Nikitenko et al. (2021)^17–19^ and references therein). However, the chemical characteristics of the spicule mineral are still under debate. In particular, there is no consensus on the chemical state of the spicule mineral. Based on XRD measurements, Odum (1951)^23^ reported that doridoidean spicules are formed of amorphous calcium carbonate. In contrast, and also based on XRD and EDS measurements, Cattaneo-Vietti et al. (1993, 1995)^5,6^ described, that the spicules consist of calcite, brucite and fluorite, hence, of crystallized mineral. Cattaneo-Vietti et al. (1995)^6^ reported that in the species that were investigated in their study, the Ca/Mg ratio of the spicules might vary, depending on their location in the organism. Ozerov et al. (2025)^13^ conducted major element analyses of the spicule material and, based on Ca/Mg ratios, identified different clusters of spicules.

The above-described studies indicate that there is still little consensus on the chemical composition as well as on the chemical state of the doridid body-wall spicules. Accordingly, the main objective of our study was an in-depth chemical and structural characterization of the spicule material. We conducted a multi-analytical approach and were checking for: (i) chemical and structural homogeneities, (ii) heterogeneities, (iii) gradation or (iv) specific structuring. In particular, we aimed to clarify the chemical state of the spicule carbonate, whether the latter is amorphous or crystalline in nature.

We investigated and describe the body-wall spicules of the doridid species *Baptodoris cinnabarina* (Bergh, 1984) (Discodorididae, Doridoidea). *Baptodoris cinnabarina* is a marine sea slug and is reported from the Mediterranean Sea and from the Atlantic Ocean. It lives between sublittoral to bathyal depths, dwells in infralittoral environments, and colonizes rocky to muddy substrates. The adult *Baptodoris* organism is 3–4 cm in length, it is very flat, has an oval and convex body and a strongly spiculated mantle, foot and body-wall^24,25^. The dorsum of *B. cinnabarina* is covered with caryophyllidia. Each caryophyllidium is supported internally by 4–7 calcareous spicules; these are oriented perpendicular to the integument surface.

With our study, we characterize structural and chemical aspects of the *B. cinnabarina* spicule material. We describe the internal structuring of the spicules, differences in chemical element composition, and address the structural state of the spicule minerals. For the latter we used a variety of analytical techniques. We present results obtained with SEM (scanning electron microscopy), TEM (transmission electron microscopy and selected area electron diffraction (SAED)) and micro-CT imaging, X-ray (XRD, SAED) and electron backscattered diffraction (EBSD) as well as results obtained from energy-dispersive X-ray (EDS) and solid state nuclear magnetic resonance (ssNMR) spectroscopy (Table 1). Our study demonstrates the amorphous nature of the *B. cinnabarina* spicule material and shows that the spicules consist of two amorphous components: amorphous calcium carbonate (ACC) and amorphous calcium phosphate (ACP). We describe the slightly graded distribution of the amorphous phases in the spicules and discuss advantages of an amorphous material for the formation of an internal scaffold.

## Materials and methods

### Materials

We investigated the spicules and the soft tissue within as well as that surrounds the spicules of up to five *Baptodoris cinnabarina* specimens (Table 1). For each specimen we investigated more than 10 spicules. The investigated spicules were from the body-wall of the selected doridid organism, however, did not separate the spicules according to their provenance within the soft tissue of the animals. All investigated specimens were collected during the INTEMARES A4 CAD expedition, in the Gulf of Cádiz, Spain.

## Methods

### Soft tissue characterization techniques

#### Fixation, preservation and embedding in methacrylate resin

Specimens selected were fixed in 4% paraformaldehyde and preserved at 4º C or, in some cases in 2.5%, glutaraldehyde solution, buffered with sodium cacodylate (0.1 M, pH 7.4). Fixation lasted for 48 h at 4 °C. The samples were preserved in cacodylate solution at 4 °C. Sample processing occurred at the Histology Unit of the Institute of Biomedicine of Málaga, Nanomedicine Platform (IBIMA Platform BIONAND). Subsequently, the samples were dehydrated and embedded in methacrylate-based resin (Technovit 7200 VLC) in five steps. The first three steps were mixtures of ethanol (Et) and increasing proportions of Technovit (T) (30T:70Et; 50T:50Et; 70T:30Et), and the last two steps consisted only of Technovit 7200 VLC. The embedded tissues were sectioned with a thickness of 50 μm using the cutting band system EXAKT 300CL. The sections were ground with a precision microgrinding system (EXAKT 400 C), stained with toluidine blue (1%), and imaged with an OLYMPUS VS120 microscope of the Central Services for Research Support (SCAI) of the University of Málaga.

### Scanning electron microscopy (SEM)

Pieces of the mantle were removed from different regions of the mantle and were fixed in glutaraldehyde. Subsequently, the samples were dehydrated in an ethanol series up to 100% ethanol. Then, samples were critical-point dried, sputter-coated with gold, and examined with a JEOL JSM-840 SEM at laboratories of the University of Málaga.

### Transmission electron microscopy (TEM)

For imaging the ultrastructure of the soft tissue that encases the spicules as well as imaging the ultrastructure of the spicules, pieces of soft tissue and mineralized spicules were fixed in a 2.5% glutaraldehyde solution, buffered with an 0.1 M sodium cacodylate solution. Fixation was at a pH of 7.4, at 4 °C and lasted for 48 h. The samples were then decalcified in a 2% EDTA solution, postfixed with OsO_4_ for 2 h at 4 °C, and stained with 2% uranyl acetate for 1 h at room temperature and in darkness. Subsequently, the samples were dehydrated in an ascending series of isopropanol (30, 50, 70, 90, 100%) and embedded in EPON or/and Spurr resin. Ultrathin Sect. (0.1 μm) were cut from the embedded samples and imaged with a FEI Talos 200X TEM at the University of Málaga, Spain, and a Thermofisher Scientific Tecnai G2 20 Twin TEM at the ICTS “NANBIOSIS” U28 unit of the Institute of Biomedicine in Málaga (IBIMA, Platform BIONAND).

### Hard tissue characterization techniques

#### Computed tomography (Micro-CT) of entire organisms

The specimens preserved in 100% ethanol were post-fixed in OsO_4_ (2%) for 1 h at room temperature. The latter improved the contrast of the soft and hard tissue. Subsequently, the specimens were dehydrated with a graded series of ethanol concentrations and were critical-point dried. The specimens were scanned with a Bruker SkyScan 2214-3D X-ray Micro-CT (voxel size = 0.36–1.1 μm), located at the Central Services for Research Support (SCAI) of the University of Málaga, and with an Xradia 510 VERSA (ZEISS) X-ray Micro-CT, located at the Centro de Instrumentación Científica (CIC) of the University of Granada. The taken micrographs were processed with the Dragonfly software.

### SEM imaging, EBSD, EDS measurements

For SEM imaging, and EBSD and EDS measurements, the spicules were embedded either into EPON resin or superglue. For obtaining even surfaces, the embedded spicules were trimmed with a trimming knife, cut with glass knives and polished with a diamond knife in an ultramicrotome. For EBSD and EDS measurements the samples were coated with 4 to 6 nm of carbon. For SEM imaging, the samples were coated with 4 nm of Pt/Pd. EBSD and EDS measurements were carried out with a Hitachi SU5000 field emission SEM, equipped with a high-resolution Nordlys II Oxford EBSD and an OXFORD X-Max80 EDS detector. During measurements, the SEM was operated at 15 and 20 kV; scans were taken with a spot size of 50 to 60. SEM imaging, EBSD, and EDS measurements were performed at the Department of Earth and Environmental Sciences, LMU Munich, Germany.

### Transmission electron microscopy (TEM), selected area electron diffraction (SAED)

Ultrathin FIB lamellae were prepared using a Thermo Fisher Helios Nanolab 650 Dual Beam System (SCAI-University of Málaga, Spain), following the standard protocol for FIB lamellae preparation. A platinum layer was initially deposited to protect the surface of the sample, and two trenches were milled at 30 kV 9 nA to isolate the area of interest. After peripheral milling, the 2 μm-thick lamella was transferred to an Omniprobe grid using a micromanipulator. Thinning was performed at 16 kV ion beam and currents from 0.15nA to 50 pA. Final low-kV cleaning was performed at 5 kV, and 120 pA. TEM images and Selected Area Electron Diffraction (SAED) patterns were taken at Max Planck Institute of Colloids and Interfaces, Germany with a double Cs corrected Jeol JEM-ARM200F TEM, operated at 200 kV and 10 µA emission current. Azimuthal integration of SAED patterns was calculated using the PASAD plugin for Digital Micrograph.

### XRD measurements

For X-ray powder diffractometry, the spicules were filled into glass capillaries with an outer diameter of 0.3 mm. The capillaries were then sealed. A capillary length of about three cm was filled for the measurements. XRD-diffractograms were recorded on a parafocussing transmission geometry, STOE STADIP diffractometer, using Mo-K_a1_ radiation (λ = 0.07093 nm) at 40 kV, 30 mA and a curved Ge (111) primary monochromator. Data sets were collected eight times in the range of 2 Theta = 10°−50° with a step size of 2 Theta = 0.01° and were added up to improve counting statistics. To allow a common representation, the diffractograms were normalized. Reference diffractograms for inorganic calcite and aragonite were taken from Liu (2014) and Pokroy (2007). XRD measurements were performed at the Department of Earth and Environmental Sciences, LMU Munich, Germany.

### ssNMR spectroscopy

The ^13^C and ^31^P NMR spectra were recorded on an AVANCE 300 Bruker spectrometer at Laboratoire de Chimie de la Matière Condensée, Sorbonne Université, Paris, France. The spectrometer was operated at 7 Tesla (ν(^1^H) = 300.13 MHz, ν(^31^P) = 121.50 MHz and ν(^13^C) = 75.51 MHz) using a 2.5 mm double channel Bruker probe at a magic angle spinning (MAS) frequency of 15 kHz. Dry *Baptodoris* spicules (about 10 mg) were inserted into 2.5 zirconia rotors and spun at a magic angle spinning (MAS) frequency of 15 kHz. Radiofrequencies were set to: ν_RF_(^1^H) = 76.2 kHz, ν_RF_(^31^P) = 87.7 MHz and ν_RF_(^13^C) = 51.8 MHz. The ^1^-^13^ C cross-polarization (CP) MAS NMR spectra were recorded using a contact time (t_CP_) of 1 or 10 ms and a recycle delay (RD) of 2.5 s. The ^31^P single pulse NMR spectra were recorded using a flip angle of 30° and RD = 60 s. The ^1^H-^13^P CP MAS NMR spectra were recorded using t_CP_ = 0.1, 1 and 10 ms and RD = 2.5 s. The two-dimensional ^1^H-^31^P heteronuclear correlation (^1^H-^31^P HetCor) NMR experiments were performed with t_CP_ = 1 ms, RD = 2.5 s, 32 t_1_ increments and 8 scans per t_1_ increments. The ^13^C and ^31^P chemical shifts were referenced (0 ppm) according to ^13^C peaks splitting of adamantine^26^.

**Table 1:**
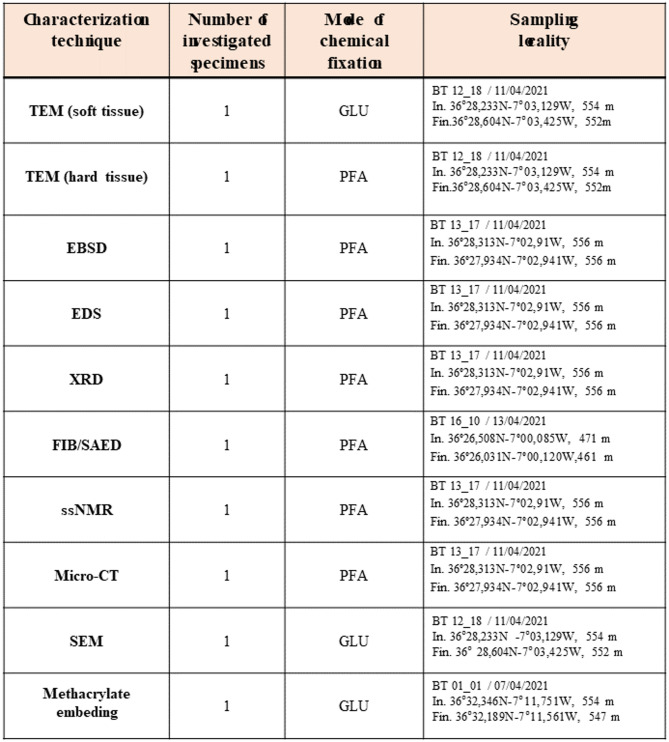
Summary of performed (i) soft and hard tissue characterization techniques, (ii) number of specimens investigated with a specific technique, (iii) mode of chemical fixation of the soft and hard tissue, (iv) geographic information on the sampling sites. TEM: Transmission Electron Microscopy, EBSD: Electron Backscatter Diffraction, EDS: Energy Dispersive X-ray Spectroscopy, XRD: X-Ray Diffraction, FIB: Focused Ion Beam, SAED: Selected Area Electron Diffraction, ssNMR: solid-state Nuclear Magnetic Resonance Spectroscopy, Micro-CT: Micro-Computed Tomography, SEM: Scanning Electron Microscopy, GLU: Glutaraldehyde, PFA: Paraformaldehyde.

## Results

Morphological and structural aspects of the spicules are shown in Figs. 1, 2, 3, 4 and 5 and S1. The amorphous nature of the spicule material is demonstrated with Figs. 5, 6 and 7. Characteristics of spicule mineral major element distributions are given in Figs. 8 and 9 and S2.

**Fig. 1:**
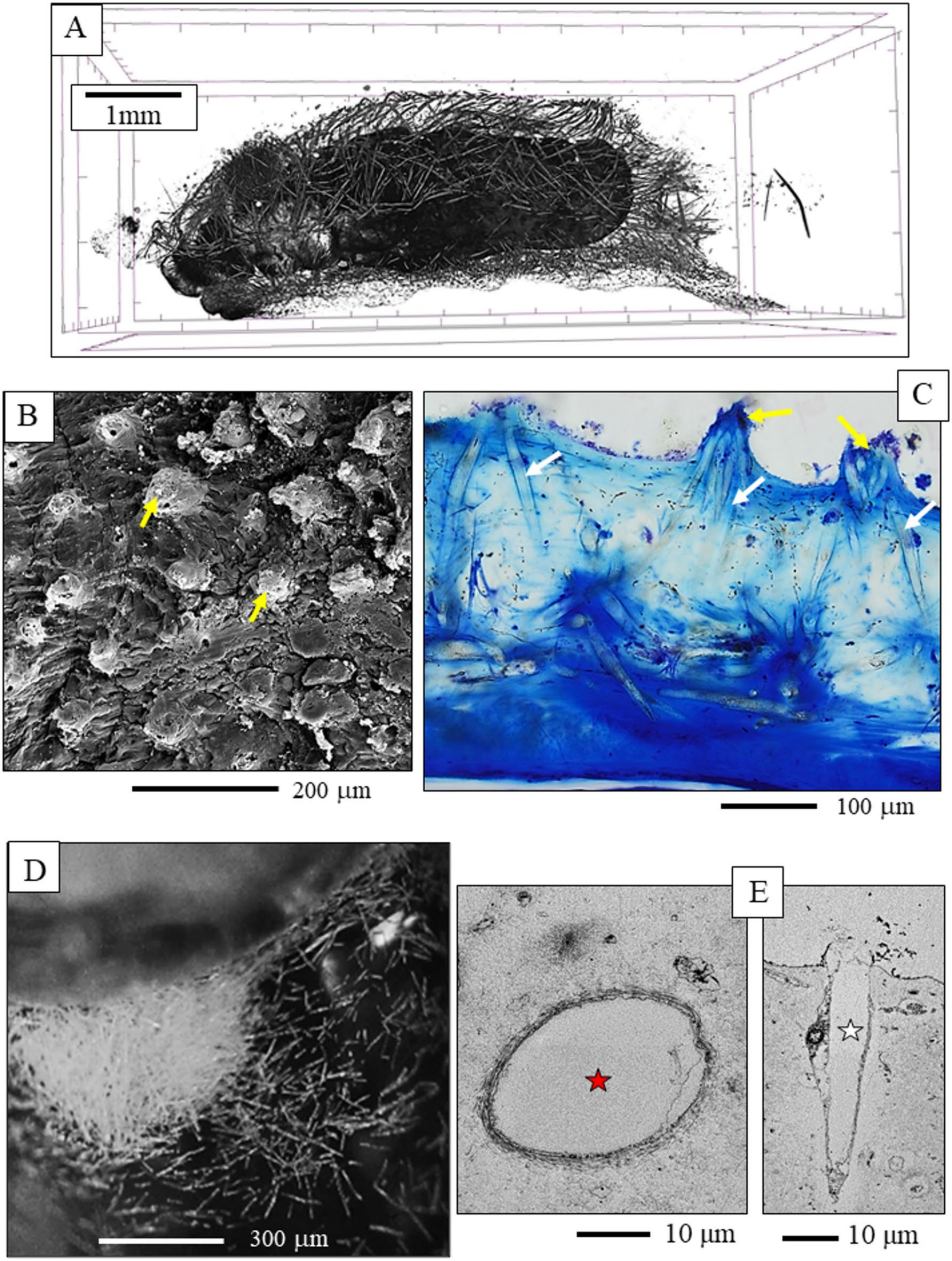
**A** to **E**: The mesodermal spicules of the *Baptodoris cinnabarina* integument. **A**: Micro-CT micrograph of a *Baptodoris cinnabarina* organism visualizing the distribution of its soft tissue and organs and the network of spicules within its integumentary system. **B**: SEM micrograph visualizing the caryophyllidia (yellow arrows), extending from the mantle surface. **C**: Optical view of a transverse section of a specimen embedded in methacrylate, showing the caryophyllidia (yellow arrows) with their assembly of vertical spicules (white arrows). These stabilize the soft tissue of individual caryophyllidia. **D**: Optical microscope image of spicules extracted from the mantle tissue. **E**: TEM micrographs of spicules cut transversely (red star in E) and longitudinally (white star in E), fully embedded into mesodermal soft tissue. Left in (E): The spicule is surrounded by muscular fibers; the black rim around the surrounding the surface of the spicule.

Figure 1A visualizes the accumulation of the spicules within the integumental soft tissue of *Baptodoris cinnabarina*. The spicules are densely placed next to each other and form a, more or less, structured network. The spicule network encases the entire organism (Fig. 1A). The assembly of spicules generates an internal scaffold and supports the mantle tissue (Fig. 1A). Well-observable is the role of the spicules at the caryophyllidia (yellow arrows in Fig. 1B, C). Here the spicules are in vertical and/or highly inclined position (white arrows in Fig. 1C) and secure, for each caryophyllidium, the vertical orientation of the caryophyllidial soft tissue (yellow arrows in Fig. 1C).

**Fig. 2:**
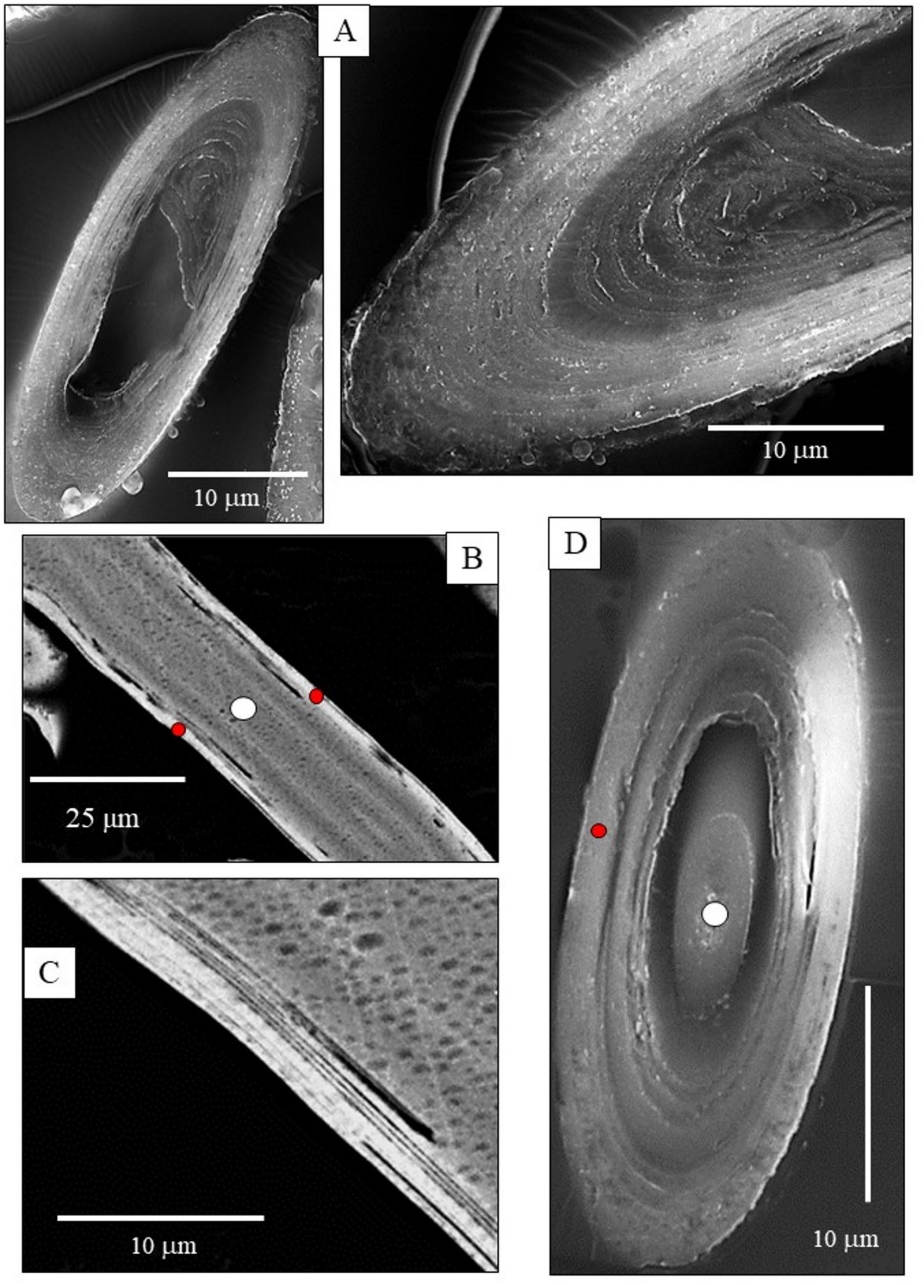
**A** to **D**: SEM images (BSE contrast) of the **B**. *cinnabarina* spicules cut in different orientations. Well observable is their internal structuring into a rim (e.g., red dots in B, D) and an internal portion (white dots in B, D). A: Note the concentric layering of the organic and the mineral substance that comprises the spicules. **C**: Note the parallel assembly of organic membranes/films that seam the spicule rim. **D**: Note the highly mineralized central portion of the spicule (white dot) and observe the difference in internal structure between the spicule rim and the central sections (C, D).

**Fig. 3:**
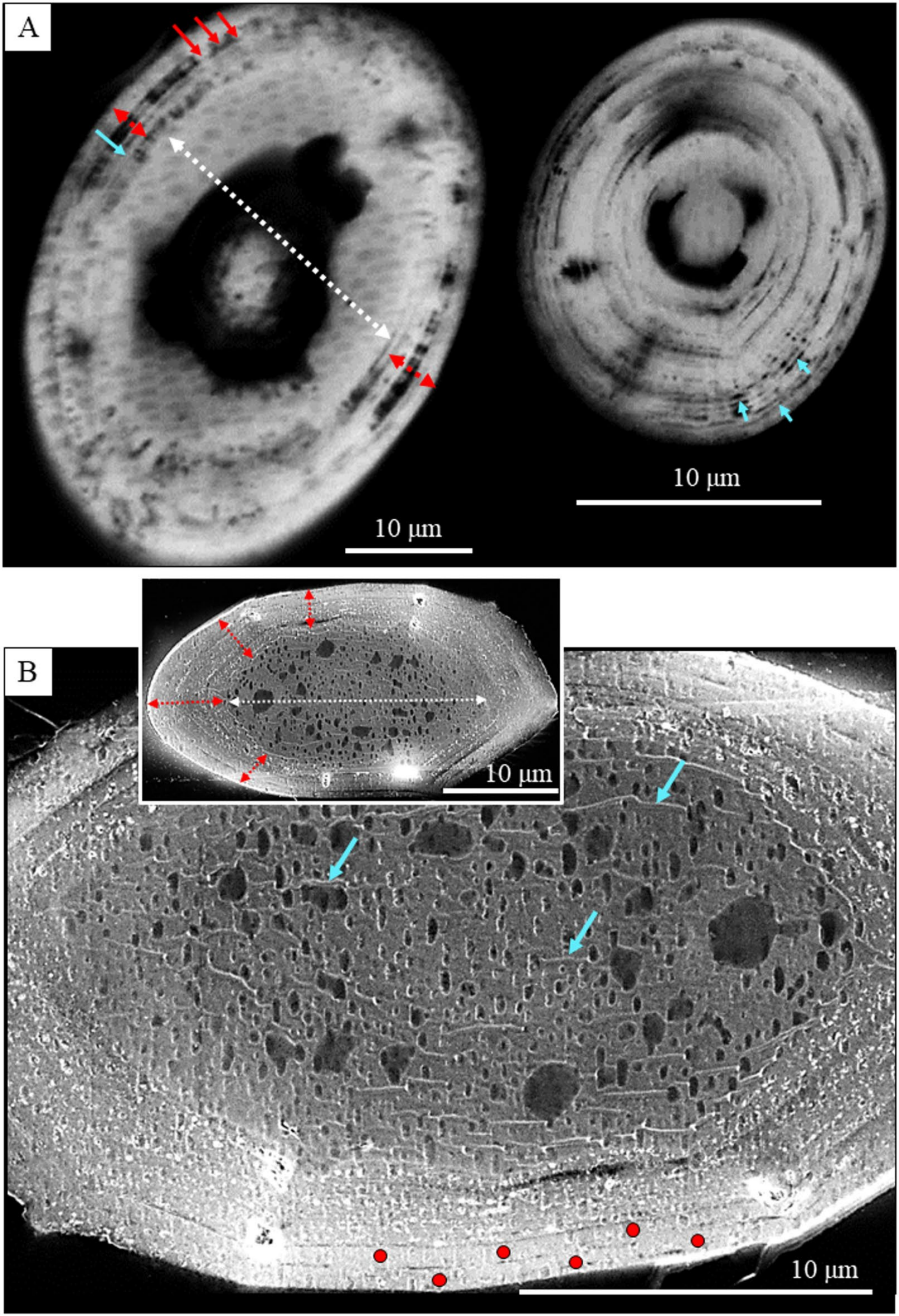
A, B: SEM micrographs (BSE contrast) that zoom-in into *B. cinnabarina* spicules. These are cut in cross-section and visualize the arrangement of organic substance and mineral within the spicules. Spicule core: Dashed white arrow in (A) and insert in (B). Spicule rim: Dashed red arrows in (**A**) and insert in (**B**). The spicule rim consists of concentrically arranged organic laminae (blue arrows in (A) and is reinforced with mineral granules (red arrows in (A)). The inner part of the spicules is poor or/and devoid of sequences of organic laminae (B) and is strongly mineralized. Nonetheless, short sections of organic films are also present within the core of the spicules (blue arrows in (B)). We observed often a sharp boundary between the rim and the core of the spicule. Red dots in (B): Concentric layers that comprise, in particular, the rim of the spicules.

Individual *B. cinnabarina* spicules (Fig. 1D) are up to 200 to 250 μm long and are about 20 to 30 μm in diameter (Fig. 1E). SEM and TEM micrographs (Figs. 1, 2, 3 and 5A to C) visualize the structure of the spicules. The spicules can be differentiated into a rim (red dots in Figs. 2B and D and 3A to C) and a core (white dots in Figs. 2B and D and 3A to C). When cut in cross-section, we observe for the entire spicule cross-section incorporation of organic lamina (Figs. 2A and 3A) and/or of thin organic layers (red dots in Fig. 3B). Along the spicule rim (red dots in Figs. 2B and C and 3A) there is a strong accumulation of organic lamina (Figs. 2B and C and 3A). Between these, we find incorporations of mineral granules (Fig. 3A). Thus, the organic substance-enriched rim is reinforced by mineral. The inner and core part of the spicules (white dot in Figs. 2D and 5A to C) appears to be more homogenous in structure and seems to be more strongly mineralized, in comparison to the spicule rim. Organic laminae are less frequent in the spicule core/center, relative to the rim (Fig. 3). Nonetheless, some organic substance is within the inner section of the spicules as well (blue arrows in Fig. 3B).

**Fig. 4:**
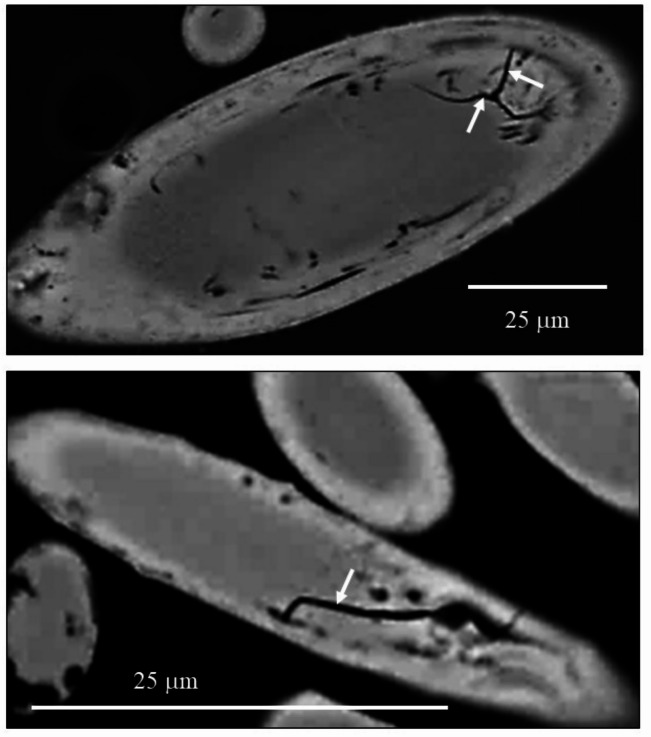
SEM images (BSE contrast) of diagonally cut spicules of *B. cinnabarina*. Note the cracks (white arrows) that form within the inner sections of the spicules and stop at their organic-rich rim.

The inner part of the spicules shows brittle failure, in contrast to the spicule rim. We observe formation of cracks that, most probably, developed at sample preparation (white arrow in the SEM micrographs of Fig. 4). The cracks stop at the organic lamina of the spicule rim.

**Fig. 5:**
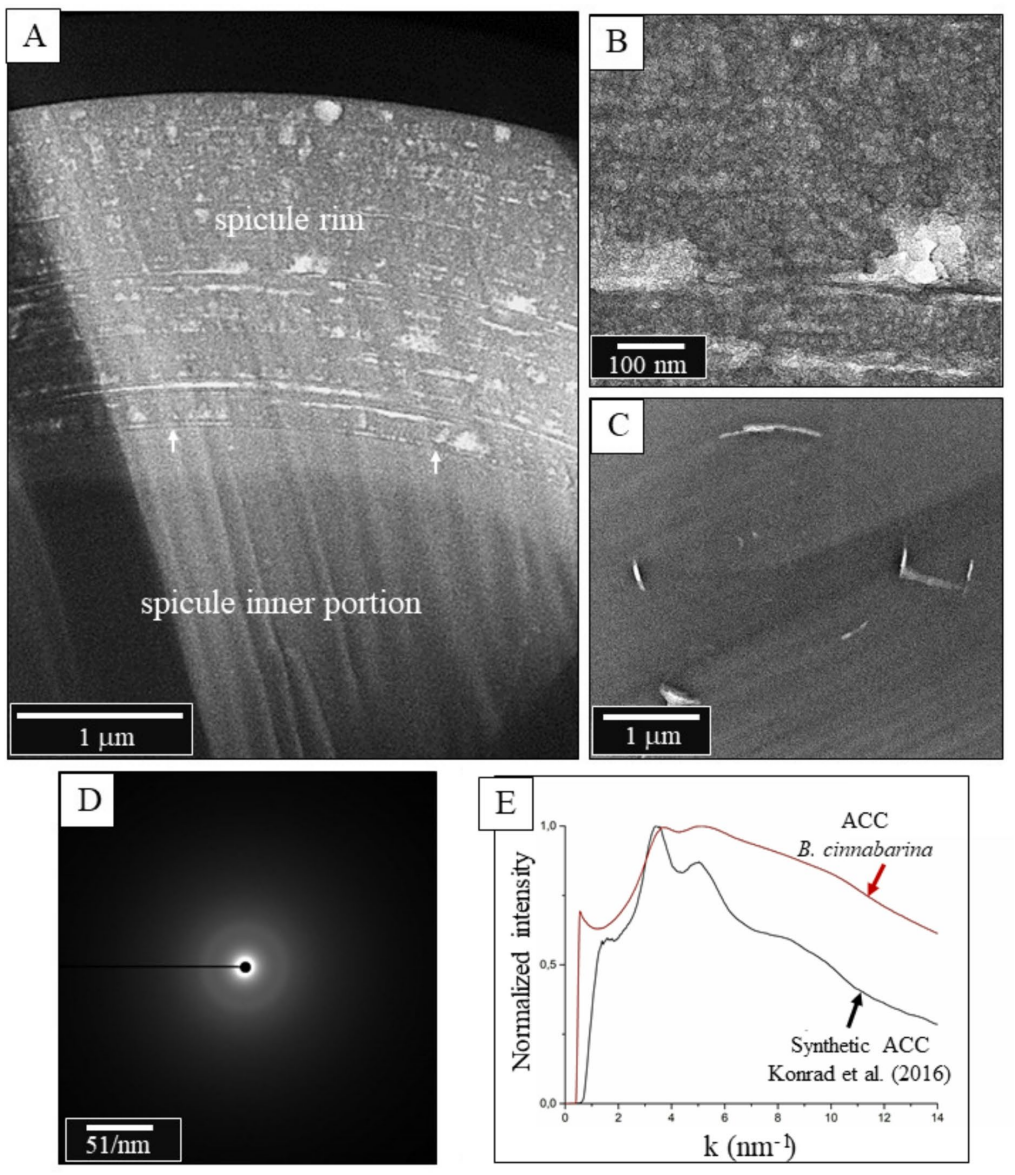
A to D: TEM and Selected Area Electron Diffraction (SAED) micrographs of the *B. cinnabarina* spicules. **A** to C: Note the difference in internal structure between the spicule rim (**B**) and the spicule core (**C**). D: SAED pattern showing only diffuse rings and indicating the amorphous nature of the spicule mineral. **E**: Comparison between *B. cinnabarina* ACC and synthetic ACC reported in Konrad et al. 2016).

**Fig. 6:**
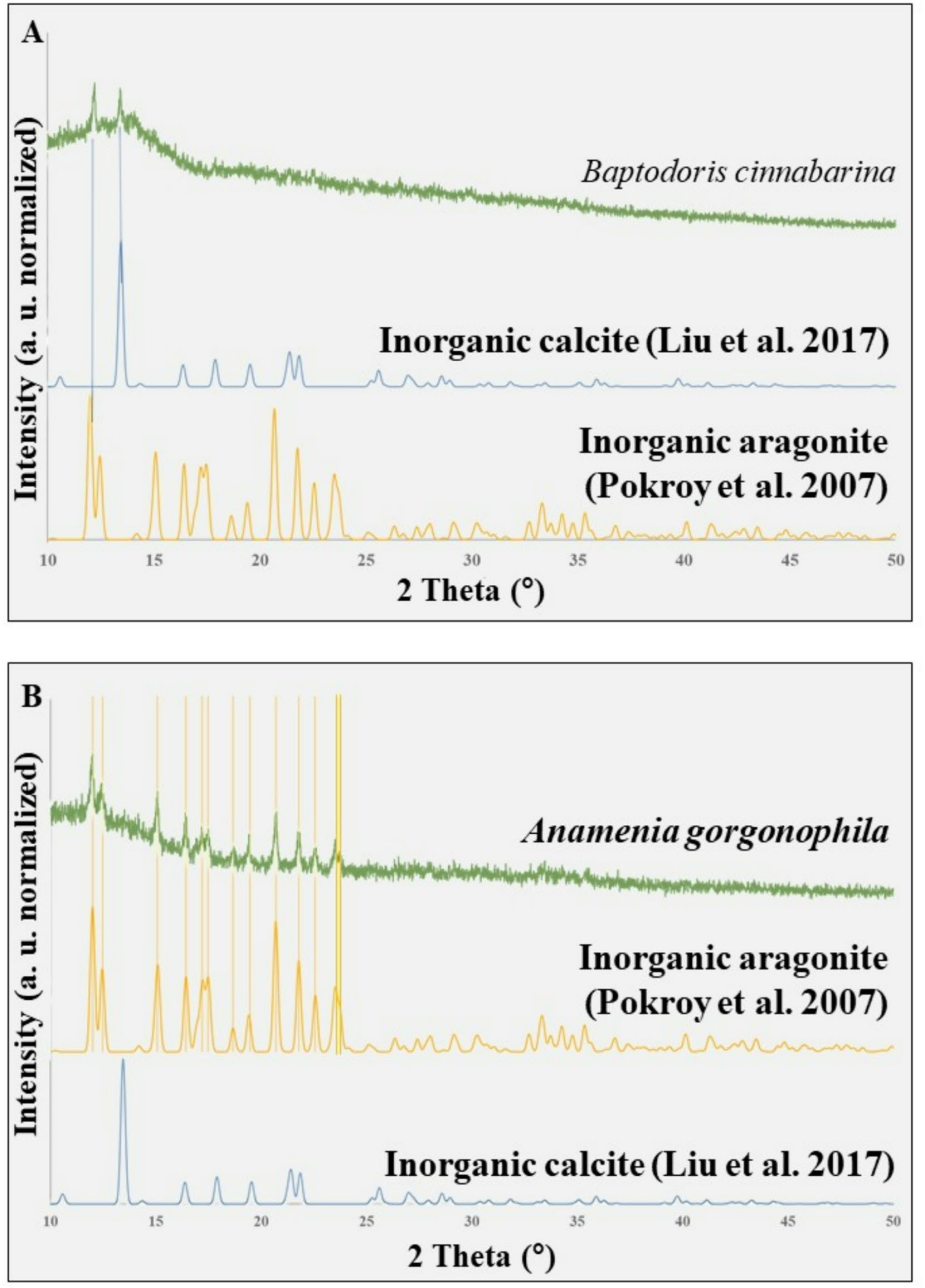
A: The amorphous nature of the calcium carbonate mineral that comprises *Baptodoris cinnabarina* spicules. A, B: XRD powder diffraction measurements: **A**: *Baptodoris cinnabarina* spicules, **B**: *Anamenia gorgonophila* Kowalevsky, 1880 spicules, Solenogastres, Mollusca (B). Both samples were measured with a capillary, as the sample holder. Well observable is that *Baptodoris* spicules consist of ACC, in contrast to the spicules of *Anamenia gorgonophila* (B), which consist of aragonite, and the inorganic reference calcite and aragonite] (B). From Liu et al. (2017) and Pokroy et al. (2007). Both molluscs form large quantities of spicules for soft tissue support and protection. However, the spicules of *Baptodoris* consist of amorphous carbonate and phosphate, while the spicules of *Anamenia* consist of crystallized Ca-carbonate (aragonite). Nonetheless, the spicules of *Baptodoris* are internal and within the soft tissue, while the spicules of *Anamenia* are external and cover the soft tissue.

**Fig. 7:**
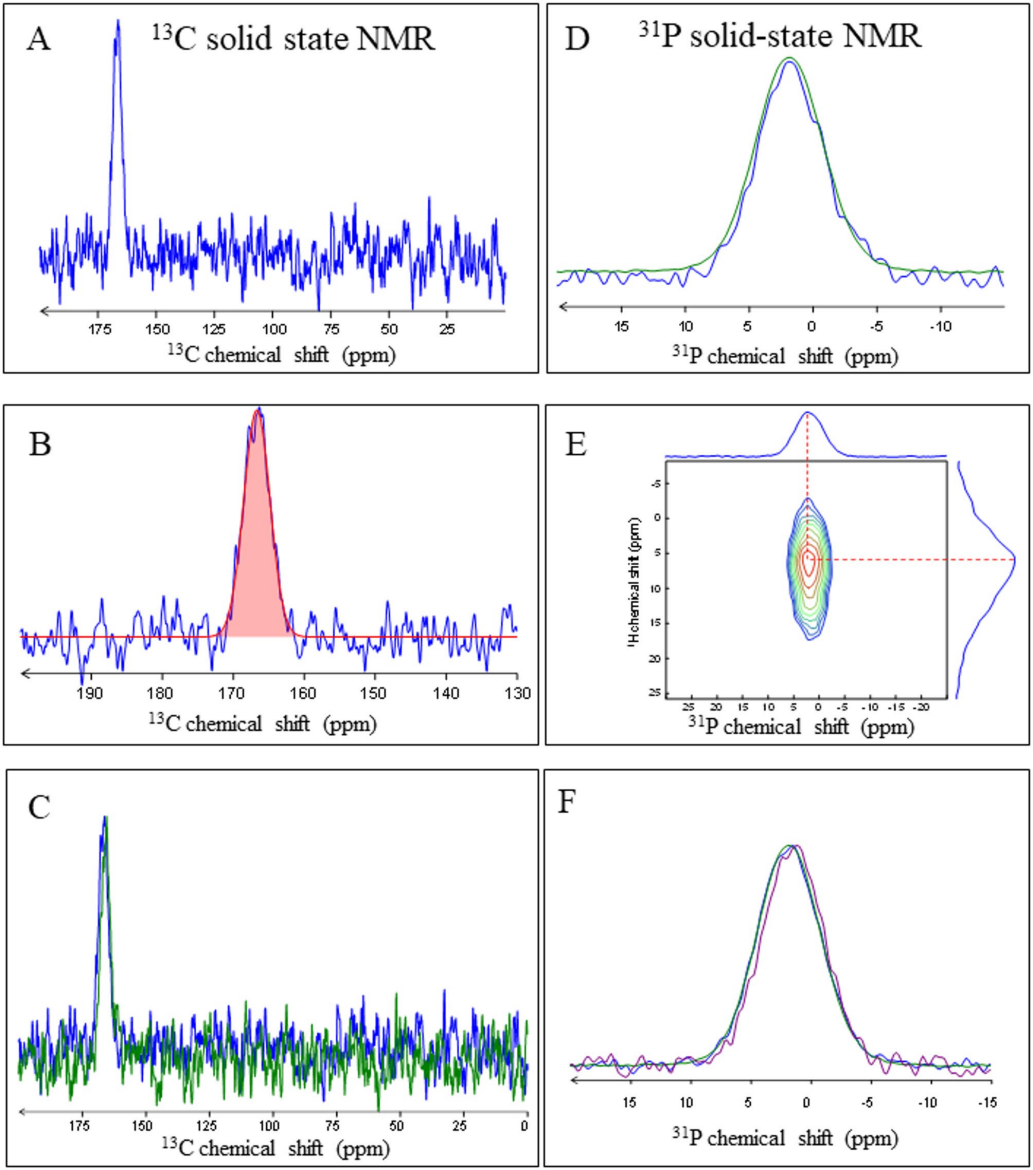
**A** to **F**: Solid state NMR results demonstrating that *B. cinnabarina* spicules consist of amorphous calcium carbonate and amorphous calcium phosphate. A to C: ^13^C; D to F: ^31^P solid state NMR results, respectively. A: ^13^C CP MAS NMR spectrum of *Baptodoris* spicules (t_CP_ = 1 ms). B: decomposition of ACC ^13^C resonance according to the following parameters: δ(^13^C) = 166.5 ppm, LW = 4.4 ppm, G/L = 1 (in red). C: ^13^C CP MAS NMR spectra of *Baptodoris* spicules recorded with t_CP_ = 1 ms (blue) and t_CP_ = 10 ms (green). D: ^31^P MAS NMR spectra of *Baptodoris* spicules recorded with direct acquisition (single pulse; blue) and with cross-polarization (^1^H-^31^P CP MAS at t_CP_ = 1 ms; green). E: Recording of a 2D ^1^H-^31^P HetCor NMR experiment, correlating phosphate and hydrogen-bearing species close in space. F: Normalized ^31^P CP MAS NMR spectra of *Baptodoris* spicules recorded with t_CP_ = 100 µs (blue), t_CP_ = 1 ms (green) and t_CP_ = 10 ms (purple). The ^13^C NMR results indicate a low amount of organic substance within the spicules and the presence of a highly disordered ACC phase. The ^31^P NMR results demonstrate the presence of ACP within the spicules. The 2D ^1^H-^31^P NMR experiment shows a single correlation resonance. The latter is particularly broad in the ^1^H dimension. This points to the presence of residual water molecules being in close interaction with the phosphate from the ACP phase.

Diffraction and spectroscopy analyses demonstrate that the spicule mineral is mainly amorphous (Figs. 5D and E, 6 and 7). Selected Area Electron Diffraction (SAED) in the TEM shows absence of sharp diffraction spots and the presence of diffuse halos (Fig. 5D), features that are characteristic for synthetic ACC (Fig. 5E). XRD measurements (Fig. 6, S1) were performed on about 2 to 3 mg of spicules. The spicules were concentrated by centrifugation to obtain a sufficient amount of material. The diffractograms show a diffuse scattering signal from the ACC, however, with minor diffraction peaks (Fig. 6). These are due to traces of calcite and aragonite. The latter are crystalline CaCO_3_ phases and result from contamination of the sample of concentrated spicules with shell fragments of other molluscs and/or foraminifera. These can be identified in the SEM (Fig. S1).

The ^13^C and ^31^P ssNMR results are shown in Fig. 7. The ^13^C ssNMR signal of inorganic carbonate is only present and has a chemical shift of 166.5 ppm. An absence of resonances in the range 0–150 ppm indicates a low amount of organic substance in the spicules (Fig. 7A, B, C). The broad ^13^C resonance peak in the carbonate region at 166,5 ppm and the gaussian shape of the peak (Fig. 7B) indicate that the spicules consist of highly disordered amorphous Ca-carbonate. When compared to other biogenic ACCs, the position of the *Baptodoris* ^13^C resonance peak is at lower chemical shift, relative to the position of the ^13^C resonance peak of other biogenic ACCs. For these, the position of the ^13^C resonance peak is between 168 and 169 ppm (see the Discussion Section), while the position of the ^13^C resonance peak of the *Baptodoris* ACC is at 166.5 ppm. The difference in ^13^C resonance peak indicates that the *Baptodoris* ACC is different in structure, relative to the ACC of other Ca-carbonate biological hard tissues.

The ^31^P resonance peak at δ(^31^P) = 1.9 ppm with a FWHM of 6.6 ppm (Fig. 7D, F) demonstrates that, in addition to ACC, a further amorphous component is present in *Baptodoris* spicules. The latter is amorphous calcium phosphate (ACP). The broadness and the perfect gaussian shape of the ^31^P resonance peak are characteristic for an amorphous phosphate component. However, in contrast to the ACC, the ^31^P resonance peak of the *Baptodoris* spicules is in the same chemical shift range as is the ^31^P resonance peak of other biogenic and abiogenic amorphous phosphate materials (see the Discussion Section). Thus, in the case of the amorphous phosphate component, we did not observe any structural difference between the ACP of the *Baptodoris* spicules and the ACP of other Ca-carbonate biological hard tissues.

The *B. cinnabarina* ACP was further characterized (Fig. 7D to F). 2D ^1^H-^31^P HetCor NMR experiments were recorded on the spicules. The 2D spectrum reveals a single correlation resonance (Fig. 7E), which is particularly broad in the ^1^H dimension and has the ^1^H intensity maximum at 5.5 ppm (Fig. 7E). This indicates the presence of residual water molecules in the spicules, interacting with the phosphate of the ACP phase. The spread to 15 ppm (Fig. 7E) highlights that protonated phosphate (e.g., HPO_4_^2-^) is present within the spicules.

**Fig. 8:**
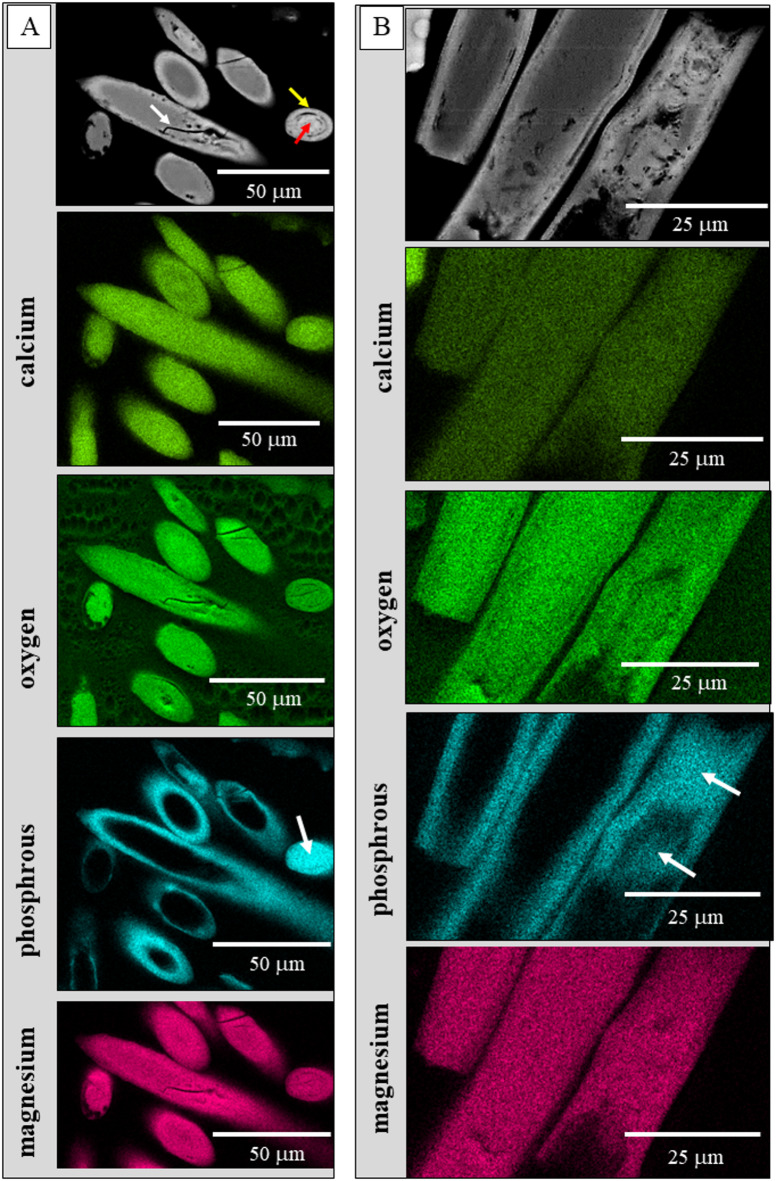
**A**, B: Major element composition of the *B. cinnabarina* spicule mineral. EDS results visualizing the distribution of calcium, oxygen, phosphorous and magnesium within the spicules. We find a clear-cut enrichment of phosphorous along spicule rims. In rare instances, we also find enrichment in phosphorous for the entire spicule (e.g., white arrows in the phosphorous maps in (**A**) and (**B**)). White arrow in the top panel of (A): See the crack within the inner part of the spicule. Red and yellow arrows in the top panel of (A) point to the well-observable difference in structure between the rim and the core. of the spicule.

**Fig. 9:**
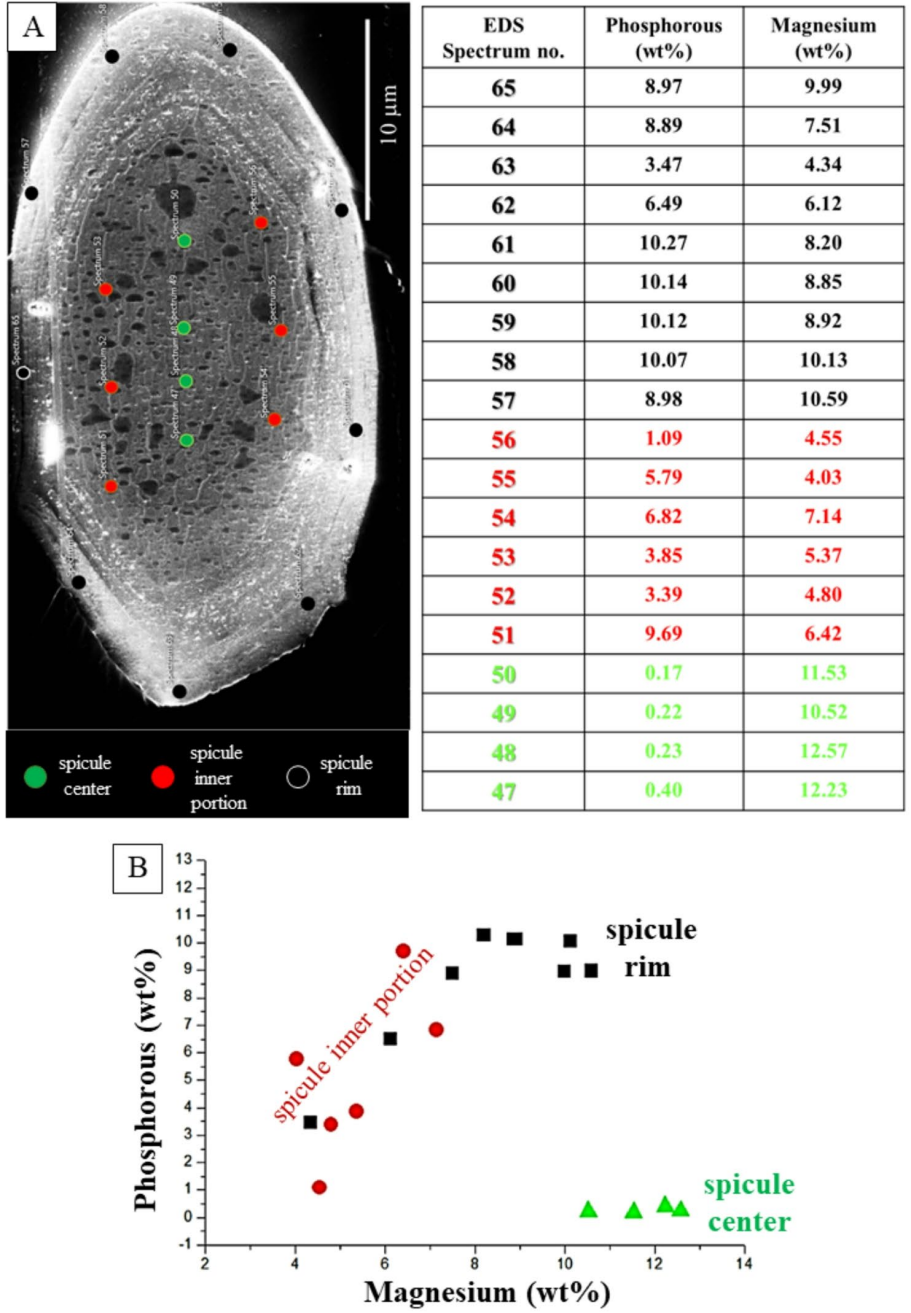
The distribution of phosphorous and magnesium concentration in different parts of a cross-sectioned *B. cinnabarina* spicule (rim, center, spicule inner portion, spicule region being intermediate between the rim and the spicule center). A: The position of EDS point-measurements and numbering of EDS spectra. **A**: Table listing for each EDS point-measurement phosphorous and magnesium concentration (wt%). Three different parts are distinguished for the spicule shown in (A): Rim (black dots and numbers in black in the table), spicule core (red dots and numbers given in red in the table), spicule center (green dots and numbers given in green in the table). **B**: Phosphorous versus magnesium concentration for the spicule shown in (A). Well-observable are the different sections of the spicules that are characterized by different P and Mg concentrations. At the rim we find high P- and high Mg-contents, while in the center we find low P-, nonetheless, high Mg contents. It should be kept in mind that organic substance is high along the spicule rim, while it is low (lower) in the spicule center (Fig. 3).

The result of EDS measurements is given in Figs. 8 and 9 and S2. Figure 8 shows EDS maps, Figs. 9, S2 highlight with phosphorous versus magnesium graphs the relation between phosphorous and magnesium contents for the different parts of the spicules, e.g., the rim and/or the core. Figures 8, S2 show the distribution of Ca^2+^, P^5+^ and Mg^2+^ for two spicules, one sectioned obliquely (Fig. 8), the other sectioned transversely but at an oblique angle (Fig. S2). We find a strong enrichment of phosphorous at spicule rims compared to the phosphorous content present at the spicule centre.

## Discussion

### The spicules

Odum (1951)^23^, Foale (1985), Foale and Willan (1987)^15,16^ and Penney (2006, 2008)^9,10^ Penney et al. (2018, 2020)^17,18^ noted that substantial amounts of mineralized spicules are incorporated into the doridoidean soft tissue. Odum (1951)^23^ reported for Archidoris that 50% of the dry organismal tissue consists of Ca-carbonate. Penney (2006, 2008)^9,10^ observed, for several doridoidean species, that up to 30% of the dry animal weight consists of Ca-carbonate. Accordingly, such a high abundance of mineralized spicules in the doridid integrument is not an occurrence by chance, neither the presence of the spicules in a subepidermal position in the mantle.

Doridid spicules vary in shape and size, their tips are not pointed, but rounded down (Figs. 2, 3, 4 and 5 in Chang et al. (2013)^27^. The distribution of the spicules in the integumental tissue does not have a random pattern, in contrast, it is structured. Micro-computed tomography scans^13,17–19^ demonstrated that the spicules form 3D networks, often with complex organizational patterns (Figs. 2, 3, 4, 5, 6 and 7 in Penney et al. (2020)^18^, Fig. 3 in Nikitenko et al. (2021)^19^, Figs. 2 and 7 in Nikitenko et al. (2025)^20^. The spicules can have a cobweb, trabecular, orthogonal or sheeted arrangement^17,18^. According to Penney et al. (2018, 2020)^17,18^, the organization of the spicule network does not vary for the different parts of a doridid mollusc organism. However, when species of different doridid genera are regarded, Kasamesiri et al. (2011)^28^ and Chang et al. (2013)^27^ reported genera-related differences of spicule arrangement. Our study confirms the results of Penney et al. (2020)^18^. We scanned the spicule network of the *B. cinnabarina* soft tissue with micro-computed tomography (CT) and did not find organizational differences for the spicules of the different parts of the animal. We also did not find any significant difference for *B. cinnabarina* spicules in spicule shape or size for the different sections of the organism.

### What is the role of the spicule assembly?

Foale and Willan (1987)^16^ were the first to demonstrate that the caryophyllidial soft tissue contains spicules, namely, each caryophyllidium 6 to 8 spicules and suggested that the principal function of the spicules is structural support of the mantle soft tissue. Thus, the role of the spicules is to keep the caryophyllidial mantle in the necessary upright position (Figs. 1, 2, 3, 4, 5 and 6 in Foale and Willan (1987)^16^; an essential requirement for the caryophyllidia to act as sensory tubercles. We observed for the spicule arrangement at *B. cinnabarina* caryophyllididia the upright position of the spicules as well (Fig. 1B, C) and propose as well that the spicules are involved in the structural stabilization of the integumental soft tissue.

Hence, the assembly of spicules can be regarded to be a flexible, supporting, internal scaffold (Figs. 9 and 10 in Penney et al. (2018)^17^, Figs. 3 and 6 in Penney et al. (2020)^18^, Nikitenko et al. (2021)^19^. Nonetheless, in addition to function as support, the spicules might also have a protective character for the organism, as a spiky soft tissue deters predators. However, it should be kept in mind that the main defensive strategy of doridid gastropods is the secretion of toxic chemicals^29,30^. Thus, the most reasonable conclusion is that the assembly of spicules in the doridid integument is an internal scaffold and a structural support for the soft tissue.

### The amorphous nature of the *B. cinnabarina* spicule mineral

For being an effective internal structural support for the fast and strongly moving doridid molluscs the spicule network has to be lightweight, rigid but also flexible to some extent. Individual spicules need to be compliant, stiff and fracture resistant. The *B. cinnabarina* spicule scaffold as well as the individual spicules allow for these requirements. Individual spicules are mineralized, however, not by crystalline, but amorphous minerals. The assembly of spicules is structured and can assume different organizational patterns, if it is necessary for the different parts of the organism.

The main results of our study are the following:

1. *The* *B. cinnabarina* *spicules are formed of amorphous mineral.* None of the used analytical techniques detected any crystallized material in *B. cinnabarina* spicules. The weak crystalline signals detected by XRD are related to contamination of the accumulated spicules with foreign carbonate materials.
2. *The largest part by volume of the* *B. cinnabarina* *spicule mineral is ACC.* However, ssNMR results show that the *B*. *cinnabarina* spicule ACC is specific, when compared to other biogenic ACCs, e.g., to the ACC of the large spine of the sea urchin *Paracentrotus lividus* (Lamarck, 1816)^32^ or to the ACC of crayfish gastroliths, e.g., the gastroliths of *Cherax quadricarinatus* (von Martens, 1868)^33^.
3. *The* *B. cinnabarina* *spicule ACC is Mg-rich*,* it is complemented with organic substance and a further amorphous mineral*,* ACP.* The amorphous phosphate is mainly present in the spicule rims. In contrast to the ACC, the ACP seems to be similar in nature to other biogenic ACPs, e.g., to the ACP of the larval shell of the gastropod *Haliotis tuberculata* (Linnaeus, 1758)^34^, or to the ACP layer of bone as well as to the ACP of biomimetic and to synthetic apatite (Wang et al. 2013)^35^. As amorphous materials usually have flexible chemical composition, Mg^2+^ is most probably randomly substituting for Ca^2+^ in the ACC. However, we cannot say with any certainty if the ACP is a dissolved component in the ACC, or whether it forms its own phase. So far, we did not find any evidence for the latter.
4. *The ACC*,* the ACP and the organic substance have a structured distribution in the spicules* (Figs. 3, 8 and 9, S2): (i) the spicule rim is enriched in ACP and organic substance, relative to the spicule core, while (ii) the spicule core is enriched in ACC and Mg. Nonetheless, the occurrences of ACP, ACC and organic substance in the spicules should not be drawn too sharply. Most likely, *there is a gradation in distribution of the ACP*,* ACC and organic substance within the spicules.*

The aforementioned findings raise two important questions:

- Why is the mineral of doridid spicules amorphous and not crystalline?
- How is the amorphous state of the mineral components stabilized?

Amorphous Ca-carbonates and Ca-phosphates are unique materials^36–40^. When formed naturally, ACC and ACP occur only as biomaterials^39,41–43^. Biological amorphous minerals/materials serve the following main functions:

1. Amorphous minerals are reported to act as precursors for the crystallized analogues^36,44–52^.
2. Amorphous minerals are used as a structural component^53–56^.
3. Amorphous minerals are reported to form temporary reservoirs for ions^56–61^. The high degree of disorder gives amorphous mineral phases a high solubility. Hence, amorphous phases are ideal for an easy mobilization of ions from the amorphous mineral deposit^62–67^.
4. The amorphous nature of the amorphous mineral renders many important material properties^41,68–70^. Due to their isotropic nature, amorphous minerals can sustain mechanical forces from all directions^36,39^. Thus, at structural material growth, amorphous mineral units have no preferred growth direction and can be shaped into any shape.
5. Amorphous minerals lack cleavage planes and are less brittle, relative to their crystallized equivalents^68–71^. It has been shown that, even though ACC and ACP have a lower hardness and elastic modulus, relative to their crystalline analogues [72, 73], ACC and ACP have an improved crack resistance, relative to the crack resistance of the crystallized counterparts^74^.

Hence, the utilization of amorphous minerals for the generation of structural hard tissues has many structural advantages.

A. Why are the *B. cinnabarina* spicules formed of amorphous minerals?

1. As crystallized phases were not detected in *B. cinnabarina* spicules, the *B. cinnabarina* ACC and ACP cannot be regarded as precursor phases for crystallized carbonate and/or phosphate.
2. At organismal growth, the spicules are not resorbed and secreted at regular intervals. Thus, the amorphous nature of the *B. cinnabarina* calcium carbonate and calcium phosphate is not for the formation of deposits and the production of crystallized tissues.
3. Biomineralized hard materials are secreted for structural and protective functions^3,4^. In the majority of cases, these materials consist of crystalline components and are used for stiffening and strengthening the surrounding tissues^3^. However, crystalline materials have the disadvantage that the crystals are anisotropic. Hence, not all crystal orientations are favourable for the generation of optimal mechanical properties^75^. In the case of amorphous minerals there is no anisotropy of material properties in, e.g., response to external load. Individual *B*. *cinnabarina* spicules have to be stiff, pliant (at least to some degree) and should not fracture easily. These properties are met with the spicule minerals being amorphous and with the spicules being coated along their rim by an array of organic lamina. The arrangement pattern and assembly density of the spicules in the scaffold give, for the scaffold as an entity, the necessary mechanical stability for the function-depending structural stabilization of the soft tissue. E. g., see the difference in spicule orientation and organization at the caryophyllidia (Fig. 1C), relative to spicule organization in other parts of the doridid mantle (Figs. 1A, 2, 3, 4, 5, 6, 7 and 8 in Penney et al. (2018)^17^, Figs. 2 and 3 in Penney et al. (2020)^18^. As stated above, spicule organization in the scaffold can assume a cobweb, trabecular, orthogonal or sheeted arrangement; as it is necessary for the spicule scaffold in the different parts of the organism^17,18^.

In summary, the amorphous state of doridinid spicule carbonate and phosphate has many structural advantages. It is very well suited as a construction material for the generation of an internal scaffold, provided that the amorphous chemical state of the spicules is well-stabilized. The *B. cinnabarina* spicule network can be addressed as a lightweight internal skeleton that unites structural support with flexibility (on the scaffold level) and stiffness and compliance (on the spicule level).

B. How is the amorphous state of the *B. cinnabarina* spicule minerals stabilized?

Our ssNMR results (Fig. 7) demonstrate that the *B. cinnabarina* spicule ACC is interspersed with ACP. EDS maps (Fig. 8) show the strong enrichment of phosphorous at the spicule rim. With EDS point measurements (Figs. 9, S2) we demonstrate the structured distribution of magnesium and phosphorous contents in the spicules. SEM images (Figs. 2 C, 3B) highlight the structured distribution of organic substance within the spicules. Accordingly, based on our results, we find for individual spicules a rather homogenous distribution of the ACC, while for the ACP, (phosphorous in EDS maps), magnesium and organic substance we observe, rather, structured distributions in the spicules (Figs. 9B, S2C). As the ^13^C CP MAS NMR experiments are highly sensitive to organic substance, due to the presence of direct C-H bonds and, as we did not detect any ^13^C organic signals when using two different contact times, we can conclude that organic substance in the spicules is present with a relatively small proportion, compared to the ACC.

Other invertebrates that mineralize, also for internal support, their soft tissue with ACC and ACP are crustaceans^63–71^. For these organisms as well, ACC forms the largest part by volume of the amorphous mineral material and the ACP is a rather minor component. For the crustaceans as well, the ACC is slightly interspersed with ACP, even though for the crustacean cuticle there is some spatial restriction of ACC and ACP. All the above-described characteristics are observed for *B. cinnabarina* spicules as well. Thus, based on the results gained in this study, and in analogy to the mineralization pattern with ACC and ACP of the crustacean cuticle^63–71,73,76^, we can assume that the ACP in *B. cinnabarina* spicules stabilizes the amorphous state of the ACC and prevents the crystallization of the ACC. The latter is supported by in vitro experiments. Studies showed that, under physiological conditions, low concentrations of phosphate prevent Ca-carbonate crystal nucleation and growth^77,78^.

However, the ACC of *B. cinnabarina* spicules is not only stabilized by the phosphate of the ACP. The latter is, in particular, the case at the spicule rim; nonetheless, in combination with the strong enrichment of organic substance at the rim (Figs. 2C and 3A). It has been debated how such a metastable phase as ACC becomes stabilised, either transitorily or permanently.

1. It has been demonstrated that low molecular weight metabolites (inorganic phosphates, phosphoenolpyruvates, citrates…) stabilize the ACC of crustaceans^62–65^. It has also been shown that specialized macromolecules in cooperation with Mg^2+^ stabilize ascidian, sea urchin and earthworm ACC^43,62,79,80^. Furthermore, it has recently been described that biomolecules stabilize residual ACC of barnacle calcite^81^.
2. Magnesium is a further ACC stabilizing agent and is widely present in the investigated spicules. It is, in particular, enriched in the central portions of the spicules, where phosphorous and organic substance are less concentrated. It has been shown that Mg heavily influences calcite nucleation and growth^82–84^.

Accordingly, at the spicule rim, the main stabilizing agent of the ACC is phosphorous, complemented by organic substance, while at spicule core and center, ACC stabilization takes mainly place with magnesium, complemented by some organic substance and some phosphorous.

In addition to stabilizing the ACC, magnesium has also an effect on the material properties of the Ca-carbonate structural hard tissue^64,84^. When magnesium is incorporated into the Ca-carbonate crystal lattice, the hardness of the crystallized Ca-carbonate structural material becomes increased. This is due to the fact that Mg^2+^ blocks the motion of dislocations^64,84^. In amorphous materials there are no dislocations. As the *B. cinnabarina* spicules consist only of amorphous mineral substance, we can assume that all the magnesium that we find in the spicules is for ACC stabilization.

We find a marked difference in structural attributes between the spicule rim and the spicule core/center (Figs. 2D and 5A to C). We often find crack development within the inner sections of the spicules (Fig. 4), but never at their rim. This indicates a difference in brittleness (lack of fracture toughness) between the spicule rim and the spicule core. As the phosphate has four bridging oxygens, it has a higher network-forming capability and a higher strengthening effect, relative to that of the ACC. The ACC has three bridging oxygens and, thus, in comparison to the phosphate, a slightly lower network-forming capability and strengthening effect. In addition, the increased fracture toughness of the spicule rim can also be attributed to the presence of concentric layers of organic substance in the spicule rim (compare Fig. 2C with Fig. 3B). These inhibit transverse crack propagation through the amorphous material of the rim. The thin layers of material, such as those at the spicule rim, have a higher bending strength, in comparison to the bending strength of the thicker layers at the spicule center and core^75^. The difference in bending strength is due to differential stress between inner and outer surface of the brittle lamina^75^.

As stated in the introduction, the formation of discrete mineralized elements/units is widespread among molluscs^85–87^. The assembly of these forms an external cover and consists of, e.g., the polyplacophoran plates, scales and spicules, or of the aplacophoran blades and spicules^89,90^. Even though being individual mineralized elements/units, the mineralized elements of the Polyplacophora and of the Aplacophora, on the one-hand-side, and the mineralized elements of the doridid Doridoidea, on the other, cannot not be regarded to be homologous structures. We find significant difference between the mineralized elements of the Polyplacophora/Aplacophora and of the doridid Doridoidea in:

(i) internal structure,
(ii) major element composition,
(iii) chemical state of the minerals,
(iv) utilization of the mineralized elements and
(v) site of formation in the mantle tissue.

It is important to recognize that the spicules of the Polyplacophora and Aplacophora and the spicules of the doridid Doridoidea are different solutions for soft tissue protection and stabilization (this study, and^88–90^. The only similarity between polyplacophoran/aplacophoran and doridoidean spicules lies in the spicular morphology of the mineralized elements.

## Conclusions

Formation of discrete mineralized elements is widespread across invertebrate phyla.

The mineralized elements of doridoidean gastropods have, by now, been described for about 150 years^22^. These are solely spicule-shaped and are fully embedded into the soft tissue of the doridid organism. The spicules form assemblies. These have specific 3D structures; however, not for the formation of an external cover but for the generation of an internal spicule framework^17–19,28^.

The mineral of the doridoidean spicule network is still little characterized and, in particular, there is no consensus about the chemical state of the spicule mineral. The aim of the presented study was to characterize with a large variety of analytical techniques the *Baptodoris cinnabarina* spicule internal structure as well as chemical state of the mineral.

We come to the following conclusions for the body-wall spicules of *B. cinnabarina*:

1. The spicules are structured composites of organic substance and amorphous mineral.
2. The spicules comprise a rim and a core with different chemical composition and internal structure. The rim is organic substance-rich and consists of arrays of organic laminae, interlaced with amorphous mineral granules. The core of the spicules is, largely, devoid of organic substance and is densely mineralized.
3. The mineral component of the *B. cinnabarina* spicule is solely amorphous.
4. *B. cinnabarina* spicules are formed of magnesium-bearing ACC, complemented with ACP. Thus, the *B. cinnabarina* spicules are composites of organic substance and two amorphous mineral components.
5. Phosphorous and magnesium contents are different for the rim and for the core of the spicules. While the spicule rim is enriched in phosphate, the core of the spicules contains no or very little phosphate. Magnesium is present in the rim, is, however, strongly concentrated in the spicule core.
6. The assembly of *B. cinnabarina* spicules can be regarded as an internal lightweight and flexible skeleton. The main role of the spicules is internal support and the stiffening of the integument, possibly supporting muscle action during locomotion.
7. Spicule aspect ratio, shape, size, orientation, and packing density in the soft tissue makes it evident that the assembly of spicules within the *B. cinnabarina* integument is for enhancement of the mechanical properties of the integument soft tissue.
8. The mineralized elements of the Polyplacophora and Aplacophora molluscs are not homologous to the mineralized elements of the doridid gastropods.

## Supplementary Information

Below is the link to the electronic supplementary material.

Supplementary Material 1

## Data Availability

The datasets generated during and/or analysed during the current study are available from the corresponding author on reasonable request.
