# Supplementary material for: Magnesium- and biopolymer-stabilized ACC and ACP form the body-wall spicules of Baptodoris cinnabarina (Doridida, Gastropoda)
Source: Sci Rep. 2026 Apr 20;16:12895. doi: 10.1038/s41598-026-47236-9 (PMC13096479; doi:10.1038/s41598-026-47236-9)
Supplement: Supplementary file 1 — Supplementary Material 1 [file 41598_2026_47236_MOESM1_ESM.docx]

**Magnesium- and biopolymer-stabilized ACC and ACP forms the body-wall spicules of *Baptodoris*** ***cinnabarina* (Doridida, Gastropoda)**

E. Griesshaber^1^, C. Salas^2^, J. D. Castro-Claros^3^, A. Sancho Vaquer^1^, E. Macías-Sánchez ^4^, X. Yin^5,1,^

G. P. Laurent^6^, N. Nassif^6^, T. Azaïs^6^, A. Checa^3^, W. W. Schmahl^1^

1. Department of Geo- and Environmental Sciences, Ludwig-Maximilians-Universität München, Munich, Germany

2. Departamento de Biología Animal, Facultad de Ciencias, Universidad de Málaga, Málaga, Spain

# 3. Departamento de Estratigrafía y Paleontología, Facultad de Ciencias, Universidad de Granada, Granada, Spain

# 4. Institute of Parasitology and Biomedicine López Neyra, Spanish National Research Council, Granada, Spain

5. Bruker, Beijing, Scientific Technology, Minhang District, Shanghai 200233, China

6. Laboratoire de Chimie de la Matière Condensée de Paris (LCMCP), Sorbonne Université, CNRS, Paris, France


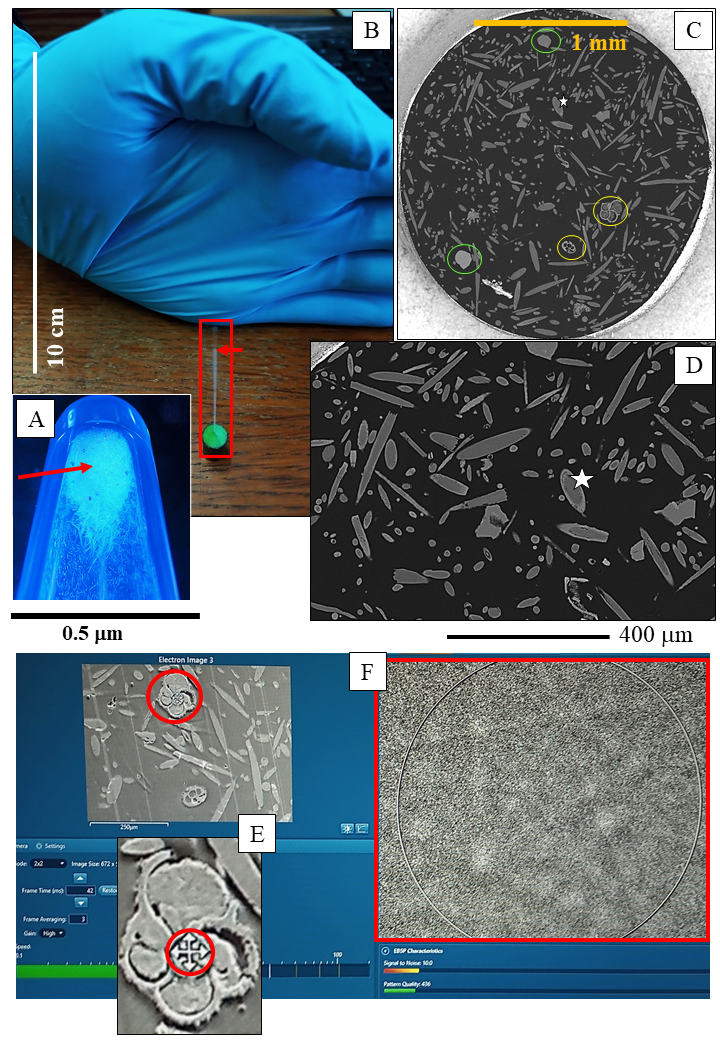


**Figure S1.** Red arrow in A: conglomeration of spicules, extracted from the soft tissue. B: A capillary, filled with spicules (red rectangle). Red arrow in B indicates up to where the capillary was filled with the spicules. C and D: Spicules embedded in EPON resin, with the surface being prepared for EBSD measurements. Impurities, in form of minute shell pieces (green circles in C) or complete shells of other marine organisms, such as foraminifera (yellow circles in C), became occasionally incorporated into the spicule conglomerate. The foraminifer shells consist of crystallized Ca-carbonate and, when scanned with the electron beam, give a Kikuchi signal (red circles and red rectangle in F).


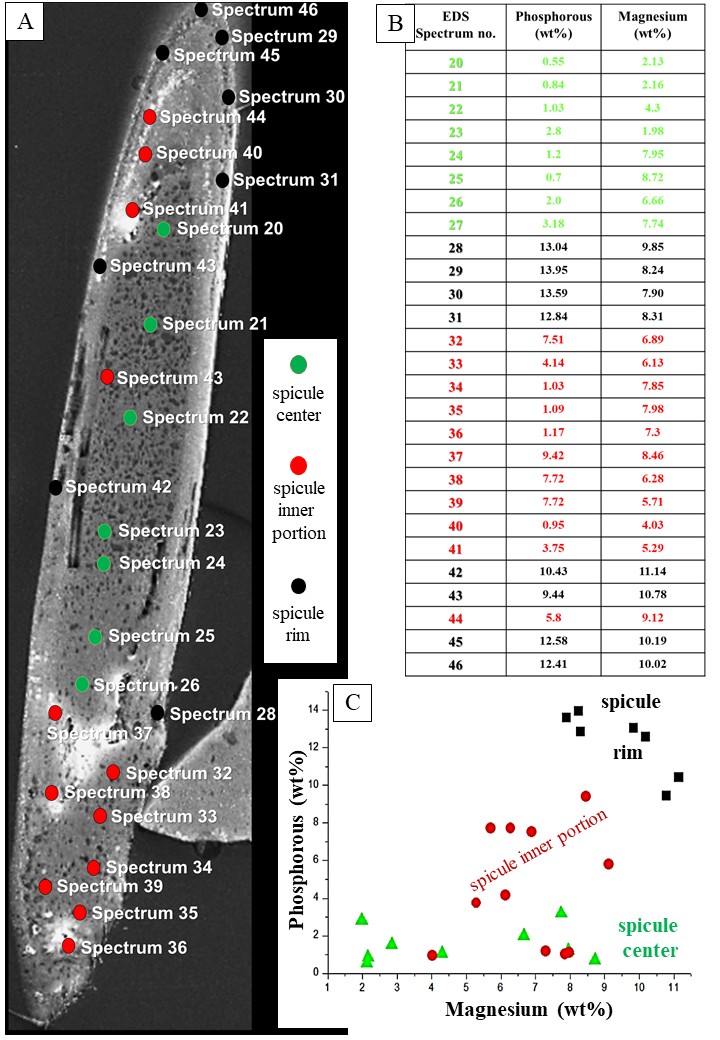


**Figure S2.** The distribution of phosphorous and magnesium concentration in different parts of a longitudinally sectioned *B. cinnabarina* spicule (rim, center, spicule inner portion, spicule region being intermediate between the rim and the spicule center). A: The position of EDS point-measurements and numbering of EDS spectra. B: Table listing for each EDS point-measurement phosphorous and magnesium concentration (wt%). Three different parts are distinguished for the spicule shown in (A): Rim (black dots and numbers in black in the table), spicule core (red dots and numbers given in red in the table), spicule center (green dots and numbers given in green in the table). C: Phosphorous versus magnesium concentration for the spicule shown in (A). We observe for the spicule rim high P- and high Mg- contents; for the spicule center low P- contents and variable Mg-concentrations.
